# Bacterial profiling of colorectal cancer biopsies: a culture-based study in Indian patients

**DOI:** 10.3389/fcimb.2025.1535477

**Published:** 2025-07-03

**Authors:** Manish Kushwaha, Shubham Chaudhary, Akhilesh Kumar Singh, Govind K. Makharia, Anil Kumar

**Affiliations:** ^1^ Gene Regulation Laboratory, National Institute of Immunology, New Delhi, India; ^2^ School of Life Sciences, Department of Biotechnology, Mahatma Gandhi Central University, Motihari, Bihar, India; ^3^ Department of Gastroenterology and Human Nutrition, All India Institute of Medical Sciences, New Delhi, India

**Keywords:** colorectal cancer, gut microbiota, culturomics, bacterial diversity, carcinogenesis

## Abstract

Emerging research has highlighted the significant role of microorganisms in the initiation and progression of colorectal cancer (CRC). However, further investigation is required to elucidate the precise mechanisms by which the microbial community or specific bacteria contribute to carcinogenesis. The present work deals with the isolation and identification of bacteria from nine CRC biopsy samples and nine adjacent normal biopsy samples. Different media, such as brain heart infusion (BHI), anaerobic basal agar (ABA), and trypticase soy agar (TSA), and culture conditions have been manipulated to maximize the isolation of bacteria residing in biopsy samples. A total of 75 bacteria were isolated from the tumor and adjacent site. *Enterococcus*, *Escherichia*, *Klebsiella*, *Shigella*, *Citrobacter, Morganella*, and *Veillonella* have been found to be enriched in most of the tumor biopsies, while biopsies collected from adjacent tissues had *Escherichia*, *Shigella*, *Enterococcus*, and *Streptococcus* bacteria. A culture-based approach to assessing bacterial diversity offer advantages, enabling the study of individual bacteria to elucidate mechanisms of intestinal carcinogenesis. This approach may provide novel insights into pathology and potentially lead to new therapeutic modalities targeting the specific bacteria implicated in the inflammation and carcinogenesis of CRC.

## Introduction

Colorectal cancer (CRC) is the third most common cancer and the second most common cause of cancer-related mortality worldwide. Despite increasing survival rates, metastatic colorectal cancer (mCRC) remains a lethal disease with a 5-year survival rate of approximately 14% (Rumpold et al., 2020). Globally, over 2 million individuals are diagnosed with CRC annually, with approximately 1 million deaths (Leowattana et al., 2023). In recent decades, increasing evidence has highlighted the presence of a unique microbiome in CRC.

The etiology of CRC is complex, involving genetic and environmental factors, with hereditary and familial CRC accounting for only 2%–5% of cases and microbiome interplay. As cancer research has advanced, the human microbiome has played a crucial role in influencing many aspects of health and disease, including the development and progression of cancer (Dekker et al., 2019). The gut microbiome has emerged as a critical player in shaping the local microenvironment of the colorectal mucosa, with potential implications for colorectal carcinogenesis. It is hypothesized that the imbalance between protective and harmful bacterial species in the gut leads to a chronic inflammation status, which could promote tumor development. While many studies have investigated the association between the gut microbiome and CRC, there is a significant gap in understanding of the specific bacterial communities implicated in CRC pathogenesis among diverse populations (Rebersek, 2021; Clay et al., 2022; Kim and Lee, 2022; Patel et al., 2022).

Recent studies have identified bacteria such as *Fusobacterium nucleatum*, *Escherichia coli*, *Enterococcus faecalis*, *Streptococcus gallolyticus*, and enterotoxigenic *Bacteroides fragilis* as closely associated with CRC carcinogenesis (Yoon et al., 2017). Therefore, characterization of the tumor microbiome is an essential step in unraveling the effects of bacteria on cancer hallmarks (Nejman et al., 2020).

A study by Dai et al. (2018) analyzed 526 metagenomic samples from diverse populations (Chinese, Austrian, American, German, and French) and identified seven bacteria enriched in CRC: *B. fragilis*, *F. nucleatum*, *Porphyromonas asaccharolytica*, *Parvimonas micra*, *Prevotella intermedia*, *Alistipes finegoldii*, and *Thermanaerovibrio acidaminovorans*. Metagenomics, the predominant method in studying gut microbial composition, faces inherent challenges such as inaccuracies in assembly results due to DNA processing variations, depth bias, incomplete genomic databases, and limited ability to detect low-abundance causative bacteria (Bilen et al., 2018). Importantly, metagenomics analysis falls short in providing live microbes for strain characterization and functional assessment (Bilen et al., 2018). These limitations hinder a complete understanding of the intestinal microbiota in healthy centenarians. Recent advancements in culturomics challenge the notion that not all microbes are culturable, demonstrating that with appropriate conditions, all microbes can be cultured (Lagier et al., 2015). Culturomics emerges as an effective complement to metagenomic sequencing for a more comprehensive characterization of gut microbial composition. Despite being recognized as a valuable approach to describe the gut microbiota (Lagier et al., 2016), culturomics and metagenomics are now seen as highly complementary techniques. Only 15% of the identified species overlap between these two methods, highlighting their synergistic potential (Dubourg et al., 2014; Diakite et al., 2019).

Metagenomics, utilizing next-generation sequencing, facilitates the direct exploration of diverse microorganisms in complex environments, surpassing the limitations of traditional microbial cultures. Despite its advantages, approximately 80% of microbial sequences identified through metagenomics remain uncultured. Additionally, this approach may not detect minor bacterial populations comprising fewer than 100,000 cells/g, for instance, the human gut microbiota, with approximately 10^12^ bacterial cells/g of stool (Eckburg et al., 2005). Metagenomics revolutionized the understanding of relations among the human microbiome, health, and diseases, but generated a countless number of sequences that have not been assigned to a known microorganism. The pure culture of prokaryotes, neglected in recent decades, remains essential to elucidating the role of these organisms (Lagier et al., 2012b).

Microorganisms can be cultivated with optimized culture tools, leading to the emergence of culturomic studies. These studies involve diverse culture conditions mimicking natural bacterial environments (Dubourg et al., 2014). In addition, culture-based methods allow for the isolation and characterization of viable bacteria, providing a comprehensive understanding of the cultivable fraction of the microbiome. This approach not only facilitates the identification of specific bacterial species but also offers insight into their functional attributes, such as antibiotic resistance and metabolic capabilities (Rettedal et al., 2014; Ito et al., 2019; Ha and Devkota, 2020; Wan et al., 2023). The recently introduced microbial culturomics is a culturing approach that uses multiple culture conditions and matrix-assisted laser desorption/ionization–time of flight and 16S rRNA for identification (Lagier et al., 2015). Before the 1980s, approximately 1,700 bacterial species had been identified. The advent of culturomics in laboratories led to the discovery of over 12,000 bacterial species. Furthermore, taxonogenomics, combining genome sequencing with traditional criteria, has facilitated the detection of novel microbial species (Lagier et al., 2015).

India, with its unique demographic, dietary, and lifestyle characteristics, presents an intriguing context for exploring the relationship between the gut microbiome and CRC. The microbiome of the Indian population is distinct from that of Western populations, exhibiting variations in microbial composition influenced by factors such as diet, cultural practices, and genetic predisposition. These differences would also be implicated in CRC (Bhattacharya, 2015; Bamola et al., 2017; Gupta et al., 2019). This study utilizes a culture-based approach to investigate the microbial composition of CRC biopsies from Indian patients, aiming to determine the prevalence and abundance of cultivable bacteria and compare the microbial profiles between tumor and adjacent normal tissues.

## Materials and methods

### Inclusion and exclusion criteria for sample collection

#### Inclusion criteria

- Age criteria—35 to 70 years for both genders.
- Participants/patients are willing to give informed consent before sample collection.
- Patients with CRC/adenoma/polyp who met the diagnostic criteria and were diagnosed as CRC/adenoma/polyp in screening methods.

#### Exclusion criteria

- Participants/patients who have used antibiotics or microecological agents within 2 months before enrolling in the research study.
- Patients who had a history were diagnosed and treated for any other form/type of cancer.
- Participants/patients who are diagnosed with any other chronic disease such as hypertension, heart, kidney, or liver disorders.
- Participants/patients who had an invasive medical intervention or undergone any surgeries in the last 3 months.
- Participants with intestinal infections or digestive tract symptoms are excluded from the research study.

### Sample collection

**Figure 1:**
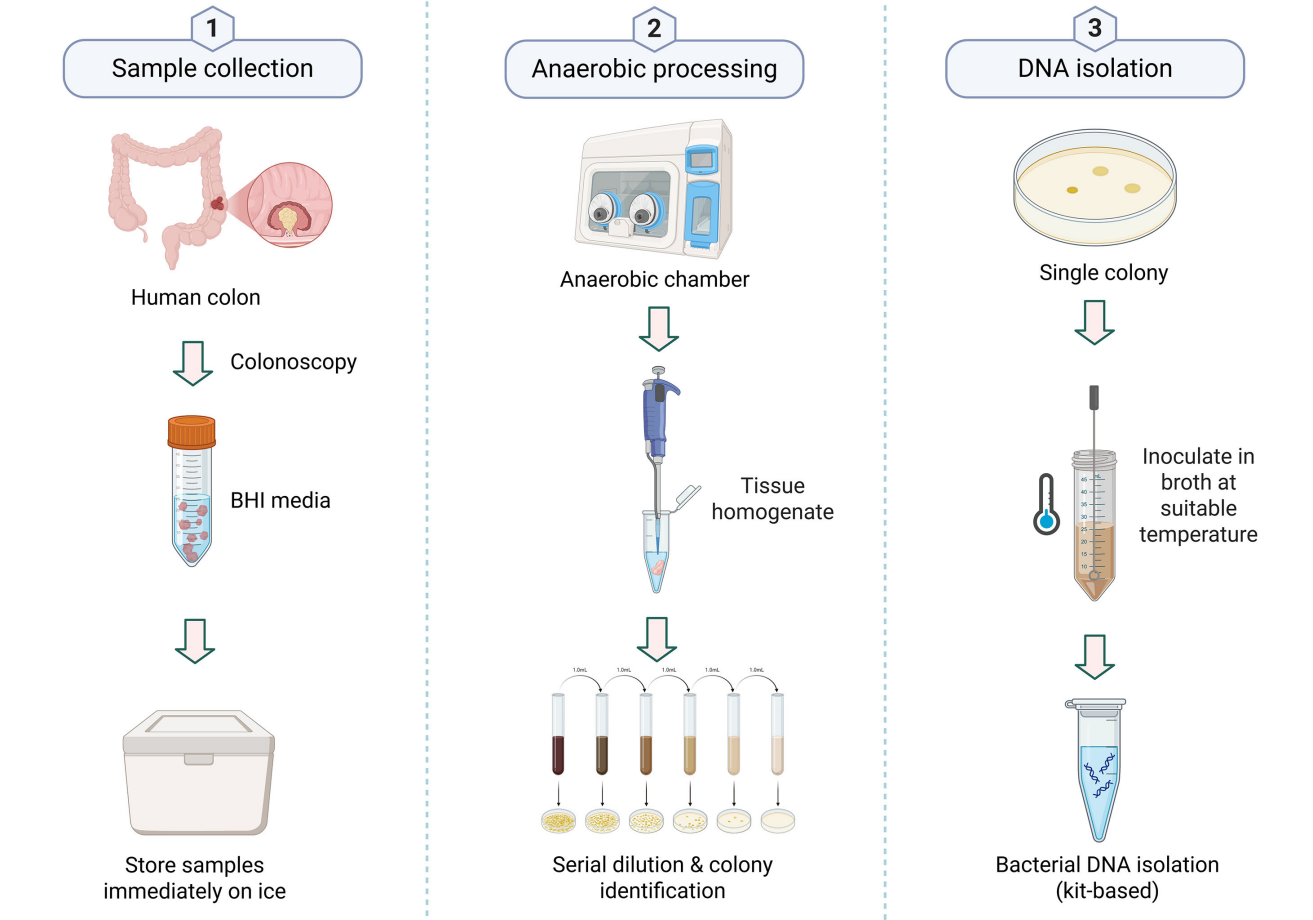
Workflow for bacterial isolation and DNA extraction from colon samples. (1) Sample collection: Tissue collected during colonoscopy is stored in BHI medium on ice. (2) Anaerobic processing: Tissue homogenization, serial dilution, and colony identification in an anaerobic chamber. (3) DNA isolation: Single colonies are cultured, and DNA is extracted using a commercial kit. Created in BioRender, https://BioRender.com/eznq0yh.

Tissue samples were collected from patients with CRC meeting the inclusion criteria at the Gastroenterology Department, All India Institute of Medical Sciences (AIIMS), New Delhi. Written informed consent was obtained from volunteers with CRC. A total of 18 colonic tissue samples were analyzed, including 9 from tumor sites and 9 from adjacent non-tumorous (normal) sites (**Supplementary Table S1**). The study cohort consisted of nine patients with CRC (five male and four female patients) aged 35 to 64 years. Histopathological analysis identified predominantly adenocarcinoma, with subtypes including signet ring cell carcinoma and extracellular mucin-producing tumors, as well as moderately and well-differentiated forms. Tumor localization was observed primarily in the rectum, with additional cases noted in the ascending colon, ileocecal junction, and hepatic flexure. The study protocol had been approved by the Institutional Bio-safety Committee (IBSC #RSG/2019/115) and the Institutional Human Ethics Committee (IHEC #113/18) of the National Institute of Immunology, New Delhi. During the colonoscopy, a biopsy was collected at the tumor site and 4–5 cm away from the tumor site, which was designated as an adjacent site. Two to three biopsy tissues were placed in separate sterile brain heart infusion (BHI) broth vials labeled for tumor and adjacent sites. The sample was transferred to ice immediately after collection (transported to ice within 30–60 min of collection) and was stored at 4°C (**Figure 1**).

### Cultivation and identification of gut bacteria using specific media

**Figure 2:**
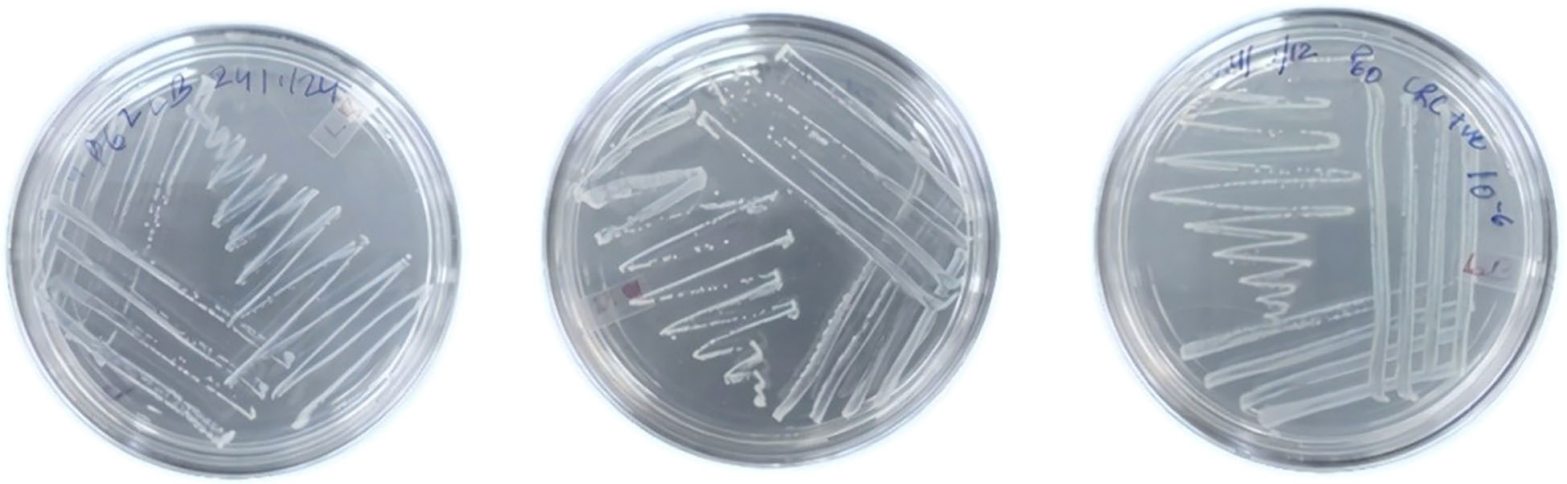
Streaking of bacterial colonies for isolation of pure cultures. Colon biopsy-derived bacteria were streaked on TSA plates and incubated at 37°C for 24–48 h. Well-isolated colonies were selected for further identification.

The isolation of bacteria was performed under anaerobic condition using a Don Whitley anaerobic workstation. In order to perform this process, biopsy samples obtained from patients with CRC were examined using both conventional isolation methods and culturomics (Lagier et al., 2012a). Biopsy were enriched in BHI media and tissues were crushed into 5 mL of media through homogenate and, after that, incubated under anaerobic condition at 37°C for 24 h. Then, bacteria were grown, using the primary culture to make serial dilution, and spread into different media plates like anaerobic basal agar (ABA), trypticase soy agar (TSA), and BHI (**Table 1**). After sub-plating until pure colonies gated (**Figure 2**), the colonies were identified on the basis of their physical appearance, morphology, shape, size, colony form, colony margin, colony texture, colony size, colony color, boundary of colonies, and fluorescence (Sousa et al., 2013).

**Table 1:** Composition of culture media used for bacterial isolation and identification.

S. no.	Media	Amount taken	Volume of Milli-Q water
1.	Anaerobic basal agar	45.9 g	1,000 mL
2.	Tryptic soya agar	45 g	1,000 mL
3.	Brain heart infusion agar	52 g	1,000 mL

### Genomic DNA extraction and preparation for 16S rRNA gene sequencing

After single-colony identification, bacteria were grown in anaerobically suitable media when they were in the log phase, and that culture was used. Genomic DNA was extracted from bacteria using the GeneJET Genomic DNA Purification Kit (Thermo Scientific) according to the manufacturer’s instructions with minor modifications (Bruggeling et al., 2021).

**Figure 3:**
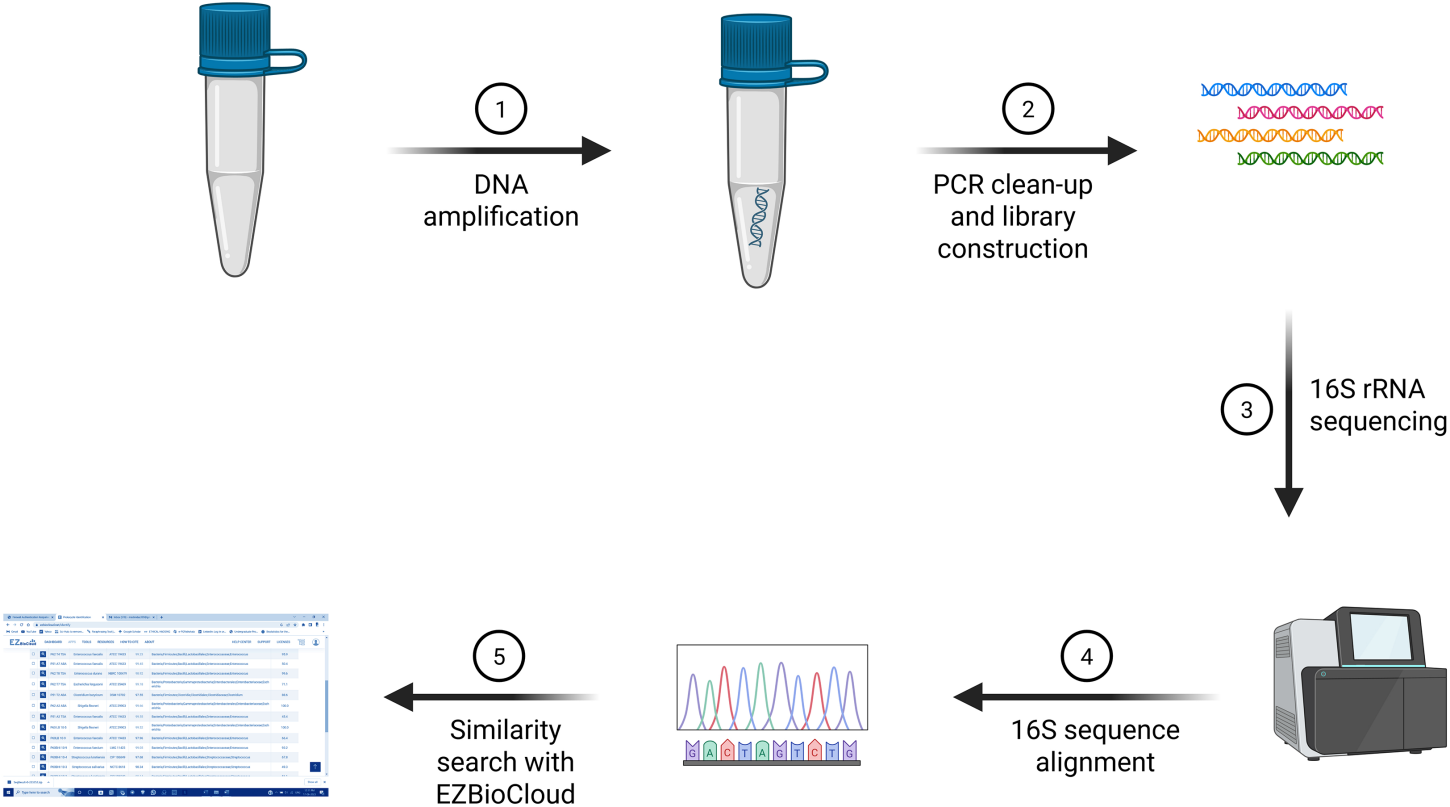
16S rRNA sequencing workflow: Genomic DNA is amplified, followed by PCR clean-up and library preparation. The 16S rRNA gene is sequenced, and the obtained sequences are subjected to alignment and taxonomic classification using the EZBioCloud database. Created in BioRender, https://BioRender.com/eznq0yh.

To harvest bacterial cells, the culture was centrifuged for 10 min at 5,000 × *g*, and the supernatant was discarded. The pellet was resuspended in 180 μL of digestion solution, 20 μL of proteinase K was added, and the mixture was incubated at 56°C for ~30 min. Then, 20 μL of RNase was added, and the mixture was incubated for 10 min at room temperature. Next, 200 μL of Lysis Solution was added, and the mixture was vortexed for 15 s. Subsequently, 400 μL of 50% ethanol was added, and the mixture was mixed. The lysate was transferred to the GeneJET Genomic DNA Purification Column, centrifuged for 1 min at 6,000 × *g*, and the flow-through was discarded. Then, 500 μL of Wash Buffer I was added, the column was centrifuged for 1 min at 8,000 × *g*, and the flow-through was discarded. Next, 500 μL of Wash Buffer II was added, and the column was centrifuged for 3 min at ≥12,000 × *g*. Optionally, the column was re-spun if residual solution was observed. The flow-through was discarded, and the column was transferred to a microcentrifuge tube. Then, 200 μL of elution buffer was added to the column; the column was incubated for 2 min at room temperature and centrifuged for 1 min at 8,000 × *g*. For a higher yield, elution was repeated with an additional 200 μL of elution buffer. The column was discarded, and DNA quantification was performed using a NanoDrop 2000 UV-Visible Spectrophotometer. The DNA was either used immediately or stored at −20°C (**Figure 3**).

### 16S rRNA gene amplification and PCR protocols

Numerous studies have utilized 16S rRNA gene sequencing to identify the composition of gut microbiota (Yatsunenko et al., 2012) (Huse et al., 2012). The 16S small subunit ribosomal gene, found exclusively in prokaryotes, served as a fundamental housekeeping gene for identifying microbial communities within samples. This gene was highly conserved but also contained hypervariable regions spanning from region V1 to region V9. Sequencing the 16S rRNA gene involved amplifying a chosen variable region through PCR using various “universal” primers, followed by sequencing (Osman et al., 2018).

**Figure 4:**
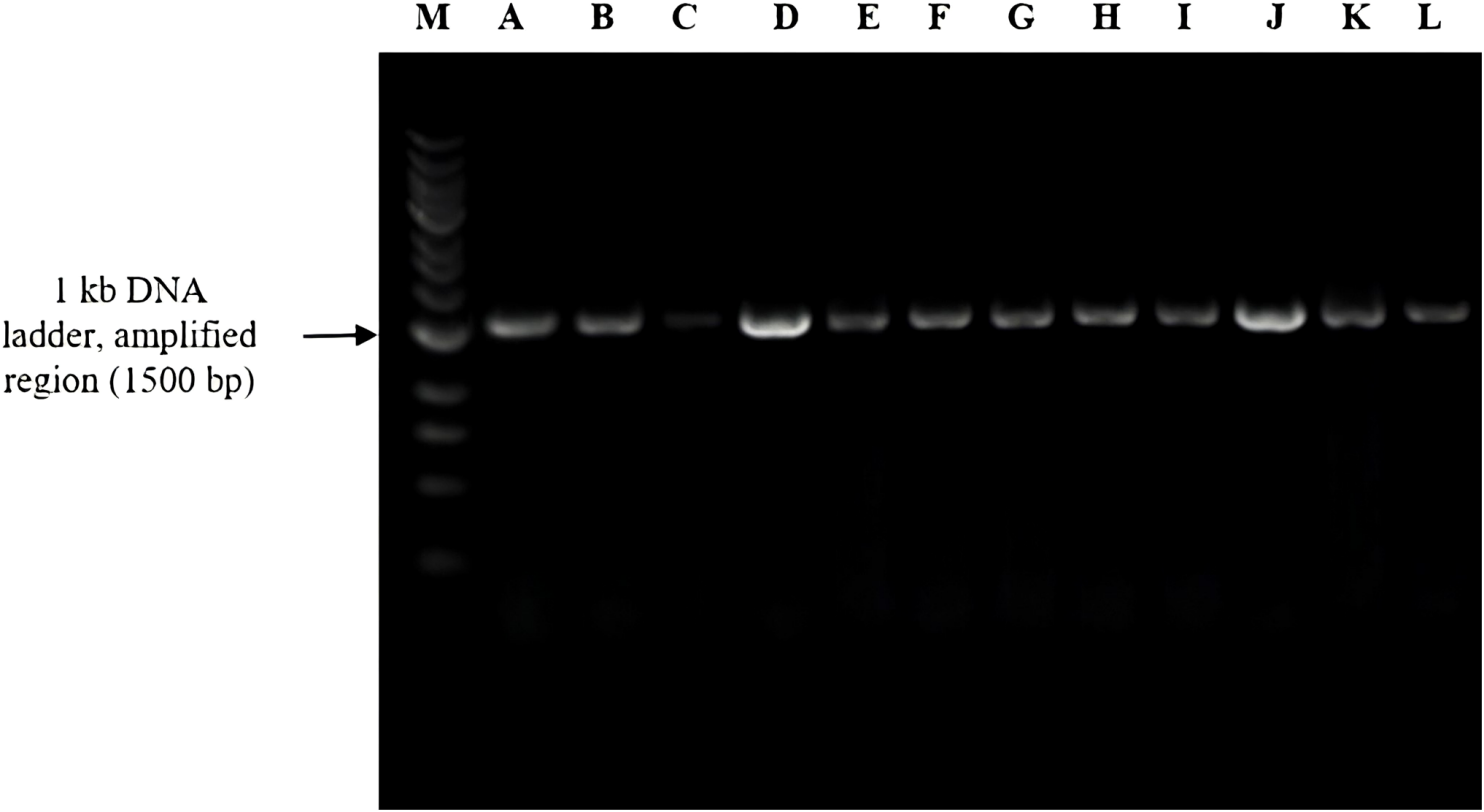
Agarose gel electrophoresis of 16S rRNA gene amplicons (~1,500 bp) from bacterial isolates labeled A–L. Lane M represents the 1-kb DNA ladder used as a molecular size reference.

The 16S rDNA samples were amplified as described earlier (Nossa et al., 2010). PCR primers used to amplify DNA included forward primer (8F) 5′-AGAGTTTGATCGTGGCTCAG-3′ 20 base pairs (bp), reverse primer (1541R) 5′-AAGGAGGTGATCCAGCCGGA-3′ 20 bp (Barcode Biosciences), and primers sent for sequencing amplified DNA: forward primer (533F) 5′-GTGCCAGCAGCCGCGGTA-3′ 19 bp and reverse primer (1100R) 5′-AGGGTTGCGCTCGTTG-3′ 16 bp (Eurofins Genomics LLC). PCR reactions were run at 95°C for 5 min, followed by 30 cycles of denaturation at 95°C for 1 min, annealing at 52°C for 1 min, and elongation at 72°C for 1 min, with final elongation at 72°C for 5 min. The PCR was performed targeting the V1–V9 hypervariable region. For PCR assays, the reaction system was 50 μL and comprised 5× PCR buffer and forward and reverse primers (2.5 μL each). DNA templates depended on the DNA concentration present in samples: dNTP (25 mM) 0.4 μL, Taq DNA polymerase 0.5 μL, and the remaining molecular-grade water. To verify the amplified DNA through DNA gel electrophoresis using the Thermo Scientific™ O’GeneRuler™ 1 kb DNA Ladder, we found that our amplified DNA sequence corresponded to 1,500 bp, as depicted in **Figure 4** alongside the ladder.

### 16S rRNA sequencing data analysis and phylogenetics

The typical length of the 16S rRNA gene, or 1.5 kb, was 1,500 nucleotides; however, different species had possessed 16S sequences that were either shorter or longer. The 16S rRNA gene was a component of the bacterial ribosome’s 30S small subunit, which bound to the Shine–Dalgarno sequence at the 3′ end (Shine and Dalgarno, 1975; Baker et al., 2003). The S1 and S21 proteins, which were known to be involved in the commencement of protein synthesis through RNA–protein cross-linking, bound to the 3′ end of 16S RNA as well (Odom et al., 1984). A single bacterium had several different 16S sequences, some of which were distinct. Numerous bacterial species featured intragenic heterogeneity, or the presence of numerous copies of the 16S gene and polymorphisms between these copies, according to genomic sequencing studies, which enabled interspecies subtyping using partial or complete 16S sequencing (Louca et al., 2018). For 16S rRNA sequencing, purified amplified DNA samples were sent to a company, which provided raw nucleotide sequence data. The sequencing reads were assembled using the DNASTAR SeqMan Pro software. The assembled sequences were subsequently analyzed using the EZBioCloud database to identify key parameters, including the closest related top-hit taxon, top-hit strain, similarity percentage, top-hit taxonomy, and genome completeness percentage (Chalita et al., 2024). Sequences that exhibited ≤98.6% similarity to reference strains were considered indicative of potentially novel bacterial species (**Figure 3**; **Supplementary Figure S1**).

### Statistical analysis

Statistical comparison of bacterial genera distribution between normal and tumor biopsies was conducted using Fisher’s exact test. The test was chosen due to the relatively small sample size (*n* = 9 per group) and the presence of categorical variables with small, expected frequencies in some of the comparison cells. Fisher’s exact test was particularly well-suited for 2 × 2 contingency tables and provided an exact probability value, which avoided approximation errors associated with chi-square testing under these conditions (Kim, 2017). For every bacterial genus, 2 × 2 contingency tables were constructed to show the absence or presence of isolates within tumor and normal biopsy groups. *p*-values were calculated to assess the statistical significance of differential genus occurrence. A *p*-value of less than 0.05 was considered statistically significant. All statistical analyses were performed using Python (version 3.10) with the SciPy library (Virtanen et al., 2020).

### Nucleotide sequence accession numbers

The raw 16S rRNA gene amplicon sequencing data have been submitted to the NCBI Sequence Read Archive (SRA) under the accession no. PRJNA1261667.

## Results

**Figure 5:**
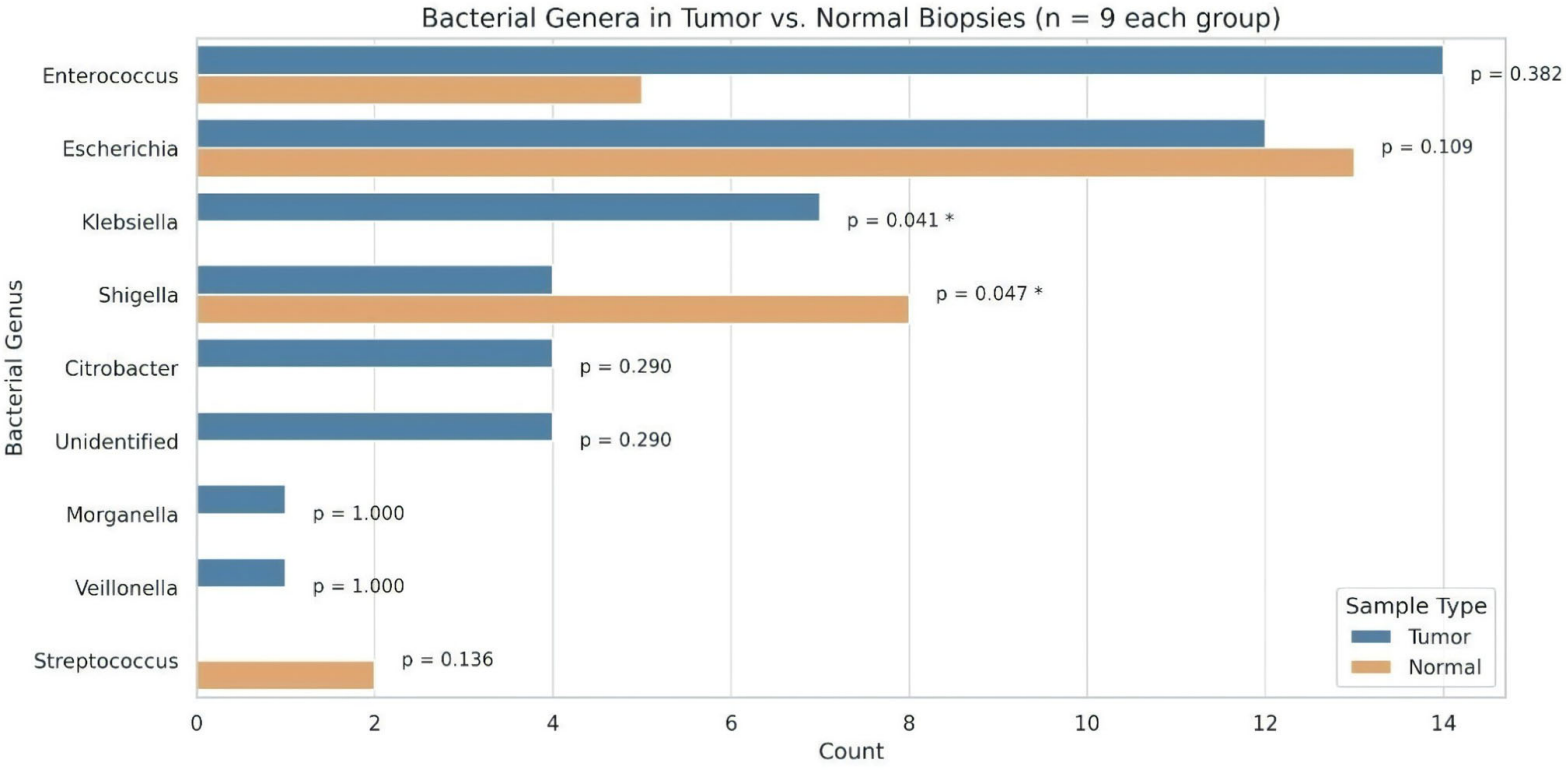
Distribution of bacterial genera identified from tumor and normal biopsy samples (*n* = 9 per group). Bar lengths represent the number of isolates per genus. Statistical comparisons between tumor and normal groups were performed using Fisher’s exact test. An asterisk (*) denotes a statistically significant difference (*p* < 0.05).

Bacterial DNA from biopsy samples, taken from both tumor and adjacent normal sites, was analyzed using 16S rRNA gene sequencing. The predominant bacterial families identified were *Enterobacteriaceae*, *Enterococcaceae*, *Streptococcaceae, Veillonellaceae*, and *Morganellaceae* present in all samples. The tumor samples showed a mix of *Enterococcaceae*, *Enterobacteriaceae*, *Streptococcaceae*, *Veillonellaceae*, and *Morganellaceae*, while adjacent non-tumor biopsies contained *Enterobacteriaceae*, *Enterococcaceae*, and *Streptococcaceae.* Interestingly, there were significant differences in bacterial profiles among individuals between tumor and control samples. Tumor tissue had a higher bacterial diversity compared to healthy tissue. Specifically, the bacteria found in tumor tissues included *Enterococcus*, *Escherichia*, *Klebsiella*, *Shigella*, *Citrobacter*, *Morganella*, and *Veillonella*, while *Streptococcus* was absent (**Figure 5**; **Table 2**). Conversely, the healthy control tissues had *Escherichia*, *Shigella*, *Enterobacter*, and *Streptococcus*, with *Klebsiella* missing. These results indicate a unique microbial composition associated with tumor tissue compared to adjacent healthy tissue. Four bacterial strains are unidentified in tumor tissues. This suggests that these bacteria might play a role in tumorigenesis or in shaping the tumor microenvironment. Understanding these microbial dynamics is crucial for unraveling the complexities of cancer development and progression.

**Table 2:** Distribution and mean abundance of bacterial genera in tumor and (adjacent) normal colorectal tissues.

Bacterial genus	Tumor isolate (*n* = 47)	Normal isolate (*n* = 28)	Total isolate (*n* = 75)
*Enterococcus*	14	5	19
*Escherichia*	12	13	25
*Klebsiella*	7	0	7
*Shigella*	4	8	12
*Citrobacter*	4	0	4
*Unidentified*	4	0	4
*Morganella*	1	0	1
*Veillonella*	1	0	1
*Streptococcus*	0	2	2
**Total**	**47**	**28**	**75**

Bold values indicate the total number of bacterial isolates identified in tumor and normal samples.

A total of 75 bacterial isolates were obtained from 18 colorectal biopsy samples (9 tumors and 9 adjacent normal tissues). Of these, 47 isolates were cultured from tumor tissues and 28 were cultured from adjacent normal tissues (**Supplementary Tables S2**, **S3**), indicating distinct microbial compositions between the groups. Most CRC cases in our cohort were localized in the rectum (**Supplementary Table S4**). *Enterococcus* was the most abundant genus in tumor (*n* = 14) and normal tissues (*n* = 5), while *Escherichia* showed a comparable distribution between tumor (*n* = 12) and normal samples (*n* = 13). *Shigella* was more prevalent in normal tissues (*n* = 8) than in tumors (*n* = 4), whereas *Klebsiella* (*n* = 7) and *Citrobacter* (*n* = 4) were detected exclusively in tumor tissues. *Streptococcus* was isolated only from normal tissues (*n* = 2), while *Morganella* and *Veillonella* (*n* = 1 each) were found solely in tumors, suggesting tumor-specific shifts in microbial colonization. The mean bacterial burden per sample was higher in tumor tissues (5.22 ± 3.67) than in adjacent normal tissues (3.11 ± 2.85), indicating potential alterations in microbial diversity associated with CRC (**Supplementary Tables S2**, **S3**).

To assess differences in the distribution of bacterial genera between tumor and adjacent normal tissues, Fisher’s exact test was applied due to the categorical nature of the data and small sample sizes. This analysis revealed statistically significant associations for specific bacterial taxa. *Klebsiella* was detected exclusively in tumor tissues (*n* = 7) and was absent in normal tissues, showing a significant association with tumor samples (*p* = 0.0412). Conversely, *Shigella* was more frequently isolated from normal tissues (*n* = 8) than tumor tissues (*n* = 4), also reaching statistical significance (*p* = 0.0468). Other genres did not demonstrate statistically significant differences in distribution. *Enterococcus* was more common in tumors (*n* = 14) than normal (*n* = 5), though this difference was not significant (*p* = 0.382). *Escherichia* showed a balanced distribution (tumor: *n* = 12; normal: *n* = 13; *p* = 0.109). Genera detected exclusively in tumor samples—*Citrobacter*, unidentified strains (*n* = 4 each), *Morganella* (*n* = 1), and *Veillonella* (*n* = 1)—also showed no significant difference (*p* ≥ 0.290). Conversely, *Streptococcus* was isolated only from normal samples (*n* = 2), but without statistical significance (*p* = 0.136).

These results indicate potential tissue-specific microbial patterns, with *Klebsiella* and *Shigella* showing statistically supported differential prevalence between tumor and normal tissues. These findings highlight distinct microbial patterns in colorectal tumor and adjacent normal tissues, warranting further investigations into their potential role in CRC pathogenesis in Indian patients.

## Discussion

The composition of the gut microbiota has a significant impact on the development of host immunity (Tomkovich and Jobin, 2016). Consequently, disruptions in microbiota composition can be harmful to the host. Interestingly, the premature infants often exhibit a high prevalence of *Enterobactericiae*, particularly *E. coli* and *Klebsiella pneumoniae*, in their intestinal microbiota (Dutta et al., 2014; Gibson et al., 2016; Chu et al., 2017).

Although the composition of microbiota in the intestinal tract can undergo changes during the initial years of life, certain bacterial strains referred to as “long-term colonizers” tend to establish stable presence. However, their persistence has been linked to the expression of various virulent genes, which could potentially harm the host. For instance, *E. coli* belonging to the B2 phylogenetic group, as noted by Nowrouzian et al. (2005), harbor the pathogenic polyketide synthase *pks* island. This genetic cluster is responsible for producing colibactin, a genotoxin capable of causing DNA damage, as elucidated by Putze et al. (2009). The presence of *pks* was strongly correlated with the majority of long-term colonizing *E. coli* strains identified in a longitudinal study involving infants, as highlighted by Nowrouzian and Oswald (2012). Our previous findings, where we demonstrated that a murine adherent-invasive *E. coli* strain (NC101) containing the *Pks* Island contributes to the pathogenesis of CRC, as reported by Arthur et al. (2012), are worth noting. Subsequent research indicated that *pks*-positive *E. coli* strains are present in the biofilm of intestinal mucosal tissues from patients with familial adenomatous polyposis (FAP). These strains, along with enterotoxic *B. fragilis*, participate in carcinogenesis in pre-clinical models, as outlined according to Dejea et al. (2018). Additionally, other bacteria such as *K. pneumoniae* have also been found to carry the *pks* gene, as documented by various studies. These bacteria exhibit cytotoxic capabilities *in vitro*, as reported by Cuevas-Ramos et al. (2010) and Lai et al. (2014).

The culture-based bacterial profiling of CRC biopsies in our study revealed a diverse array of bacterial species present within the tumor microenvironment. The identification of specific bacterial strains, such as *E. faecalis* and *E. coli*, suggests a potential association with CRC. A number of studies have reported that bacteria are associated with CRC (Li et al., 2022b). Additionally, *K. pneumoniae* was detected in tumor biopsies, potentially explaining genetic similarities with *E. coli.* If you see our results, which show more bacterial presence in the tumor site compared to adjacent biopsies, there are also bacterial species differences in both. We have analyzed a total of 18 biopsies, with 9 tumor biopsies having identified 47 bacteria and 9 adjacent biopsies having identified 28 bacteria. Bamola et al.’s study on the metagenomics of patients with CRC indicated a notable increase in the abundance of phylum *Firmicutes* in both experimental groups (Bamola et al., 2022).

Secondary bile acids (SBAs), such as deoxycholic acid (DCA), produced by gut microbiota, are implicated as oncometabolites in CRC, with their aberrant accumulation frequently reported in clinical studies (Tian et al., 2016; Yusof et al., 2018; Lin et al., 2019). This accumulation drives tumorigenesis, as DCA induces oxidative DNA damage, mitochondrial dysfunction, and genomic instability (Payne et al., 2008), which, in turn, activates proliferative pathways like EGFR/Ras/MAPK and PI3K/Akt/NF-κB (Im and Martinez, 2004; Lee et al., 2010). Alongside SBAs, polyamines, particularly N1,N12-diacetylspermine, are elevated in patients with CRC, further promoting tumor progression through related metabolic disruptions (Venäläinen et al., 2018). However, not all SBAs are oncogenic; ursodeoxycholic acid (UDCA) and lithocholic acid (LCA) counteract inflammation, reducing colonic damage and suggesting a protective role (Ward et al., 2017). These contrasting effects tie into diet, where excessive red meat and fat intake generates trimethylamine N-oxide (TMAO), a microbial metabolite linked to increased CRC risk (Xu et al., 2015).

Building on microbial influences, our study found elevated *Citrobacter*, *Morganella morganii*, and *Veillonella* at tumor sites, alongside *Klebsiella* and *Enterococcus*, indicating localized dysbiosis. Specifically, *M. morganii* exacerbates this environment by producing indolimines, genotoxic metabolites that damage DNA and accelerate CRC in preclinical models (Cao et al., 2022; Leake, 2022). Similarly, *Citrobacter* spp., often tied to polymicrobial infections and cancer comorbidities, likely amplify inflammation at these sites (Dziri et al., 2022). *Veillonella* spp., typically normal flora, may also contribute, as their rare association with CRC bacteremia suggests a role in dysbiosis-driven malignancy (Karki et al., 2023). Collectively, these findings align with broader evidence that gut microbial metabolites, including SBAs and hydrogen sulfide, promote tumorigenesis by modulating inflammation and DNA integrity, in contrast to protective short-chain fatty acids (Louis et al., 2014).

Bacteria contribute to CRC through chronic inflammation, genotoxicity, metabolic dysregulation, immune evasion, and intestinal barrier disruption (Chen et al., 2017). Pathogens like *F. nucleatum* and *B. fragilis* activate immune responses via Toll-like and NOD-like receptors, triggering NF-κB signaling and pro-inflammatory cytokine release, promoting tumorigenesis (Kaplan et al., 2009, 2014). Genotoxic bacteria, such as *E. coli* (*pks+*), produce colibactin, inducing DNA damage and mutations in tumor suppressor genes, while *B. fragilis* secretes toxins activating Wnt/β-catenin signaling (Li et al., 2022a; Joo et al., 2024). Microbial metabolism further drives cancer progression through oncometabolites like SBAs (e.g., DCA) and polyamines, enhancing cell proliferation. *F. nucleatum* facilitates immune evasion by inhibiting cytotoxic T cells through FadA adhesin and CEACAM1 interactions (Galaski et al., 2021). Dysbiosis weakens the colonic barrier, increasing bacterial translocation and epithelial dysfunction, fueling neoplastic transformation. Understanding these mechanisms may aid in developing microbiome-targeted CRC prevention and treatment strategies. While our findings offer valuable insights, we acknowledge that a formal power calculation was not performed due to the exploratory nature of the study and limited biopsy availability. Future studies with larger cohorts and prior power analysis are recommended to strengthen and validate these observations.

## Conclusion

Microbial culturomics represents a cutting-edge culture method aiming to replicate natural microbial ecosystems. This technique underscores the importance of meticulously controlled anaerobic conditions for accurately characterizing a representative portion of human bacteria, crucial for extrapolating results from extensive gut microbiome studies. Notably, variations in microbial composition have been observed between biopsy tissue samples and adjacent tumor samples, highlighting a distinct adherent bacterial population. Rigorous anaerobic culture is pivotal for advancing culturomics, a technology poised to significantly enhance our understanding of specific interactions between the gut microbiota and individual human hosts. Serving as a critical complement to metagenomic sequencing techniques, culturomics offers a broad detection range encompassing all living eubacteria and archaea. At the heart of culturomics studies lies MALDI-TOF mass spectrometry, providing a swift, accurate, and cost-effective means for microbial identification. While pyrosequencing remains relevant, particularly in scenarios where MALDI-TOF faces limitations, culturomics leverages diverse approaches in environmental microbiology, tapping into this extensive and renewable resource for clinical microbiology. Delving into the extensive repertoire of the human gut microbiome enabled by culturomics poses a significant challenge, necessitating high-throughput enriched media with selective materials to isolate minor microbial communities. Furthermore, combining culturomics with taxonogenomics for identifying novel microbial species is poised to shape the future of microbiological research. Additionally, applying culturomics to the human gut microbiota holds promise for bacteriotherapy in inflammatory bowel diseases and as an immunomodulatory approach for cancer therapy. Moreover, culturomics serves as a valuable resource for discovering new antibacterial agents and gaining insights into antibacterial resistance genes.

## Data Availability

The datasets presented in this study can be found in online repositories. The names of the repository/repositories and accession number(s) can be found in the article/**Supplementary Material**.
