# Supplementary material for: Bacterial profiling of colorectal cancer biopsies: a culture-based study in Indian patients
Source: Front Cell Infect Microbiol. 2025 Jul 3;15:1535477. doi: 10.3389/fcimb.2025.1535477 (PMC12267230; doi:10.3389/fcimb.2025.1535477)
Supplement: Supplementary file 1 [file Table1.docx]

**Culture-based bacterial profiling of colorectal cancer biopsies from Indian patients**

Manish Kushwaha^1, 2^, Shubham Chaudhary^3^, Akhilesh Kumar Singh^2^, Govind K. Makharia^3^, Anil Kumar^1,^ *

**Supplementary Figures and Table**

| **Sample ID** | **CRC/Adjacent biopsy** |
| --- | --- |
| P1, P2, P3, P4, P5, P6, P7, P8 and P9 | Colorectal cancer biopsy (47) |
| A1, A2, A3, A4, A5, A6, A7, A8, and A9 | Adjacent biopsy (28) |

**TABLE S1** List of bacterial isolates from tumor tissue and adjacent tissue. 47 bacterial isolates were obtained from nine different tumor biopsy samples, while 28 bacterial isolates were isolated from nine adjacent biopsy samples.

**TABLE S2** List of bacteria isolated from a tumor biopsy sample. Classification includes domain, phylum, class, order, family, and genera. Similarity% shows the resemblance of the sequence with the nearest relative present in the taxonomically united database of 16S rRNA gene sequences (Ezbiocloud.com). ATCC no. represents the top hit strain reference

| **S.No** | **Sample** | **Identification** | **Similarity** | **Top hit** |
| --- | --- | --- | --- | --- |
|  | **P1** |  |  |  |
| 1. |  | *Klebsiella pneumoniae subsp. ozaenae* | 99.79 | ATCC 11296 |
| 2. |  | *Enterococcus faecalis* | 99.27 | ATCC 19433 |
| 3. |  | *Enterococcus gallinarum* | 97.61 | NBRC 100675 |
| 4. |  | *Enterococcus gallinarum* | 99.73 | NBRC 100675 |
| 5. |  | *Enterococcus durans* | 99.10 | NBRC 100479 |
| 6. |  | *Citrobacter portucalensis* | 99.86 | A60 |
| 7. |  | *Veillonella atypica* | 99.95 | KON |
| 8. |  | *Citrobacter portucalensis* | 99.25 | A60 |
| 9. |  | *Klebsiella huaxiensis* | 99.86 | WCHKl090001 |
| 10. |  | *Klebsiella pneumoniae subsp. ozaenae* | 98.52 | ATCC 11296 |
| 11. |  | *Morganella morganii subsp. sibonii* | 99.8 | DSM 14850 |
| 12. |  | [*Citrobacter freundii*](https://www.ezbiocloud.net/taxonomy?tn=Citrobacter%20freundii) | 99.73 | DSM 30039(T) |
|  | **P2** |  |  |  |
| 13. |  | *Klebsiella pneumoniae subsp. ozaenae* | 99.79 | ATCC 11296 |
| 14. |  | *Citrobacter portucalensis* | 99.73 | A60 |
| 15. |  | *Klebsiella pneumoniae subsp. ozaenae* | 99.79 | ATCC 11296 |
| 16. |  | *Escherichia marmotae* | 99.45 | HT073016 |
| 17 |  | *Escherichia marmotae* | 99.25 | HT073016 |
| 18. |  | *CP040443_s 93* | 99.12 | E4742 |
| 19. |  | [*Escherichia ruysiae*](https://www.ezbiocloud.net/taxonomy?tn=Escherichia%20ruysiae) | 99.25 | OPT1704(T) |
| 20. |  | [*Klebsiella quasipneumoniae subsp. quasipneumoniae*](https://www.ezbiocloud.net/taxonomy?tn=Klebsiella%20quasipneumoniae%20subsp.%20quasipneumoniae) | 99.72 | [HG933296](https://www.ezbiocloud.net/16SrRNA?ac=HG933296) |
| 21 |  | [*Klebsiella variicola subsp. variicola*](https://www.ezbiocloud.net/taxonomy?tn=Klebsiella%20variicola%20subsp.%20variicola) | 99.52 | DSM 15968(T) |
| 22. |  | *Escherichia marmotae* | 99.31 | HT073016 |
|  | **P3** |  |  |  |
| 23. |  | *Enterococcus faecium* | 99.59 | LMG 11423 |
| 24. |  | *Enterococcus durans* | 99.66 | NBRC 100479 |
| 25. |  | *CP040443_s* | 99.45 | E4742 |
| 26. |  | [*LFHY_s*](https://www.ezbiocloud.net/taxonomy?tn=LFHY_s) | 99.32 | B1147 |
| 27. |  | *Escherichia marmotae* | 98.40 | HT073016 |
| 28. |  | [*Escherichia fergusonii*](https://www.ezbiocloud.net/taxonomy?tn=Escherichia%20fergusonii) | 99.11 | ATCC 35469(T) |
| 29. |  | [*Shigella flexneri*](https://www.ezbiocloud.net/taxonomy?tn=Shigella%20flexneri) | 99.18 | ATCC 29903(T) |
| 30. |  | *Escherichia marmotae* | 99.39 | HT073016 |
|  | **P4** |  |  |  |
| 31. |  | *Enterococcus faecalis* | 99.93 | ATCC 19433 |
| 32. |  | *Enterococcus faecium* | 99.73 | LMG 11423 |
| 33. |  | *CP040443_s* | 99.32 | E4742 |
|  | **P5** |  |  |  |
| 34. |  | *Enterococcus faecalis* | 98.9 | ATCC 19433 |
| 35. |  | *Escherichia fergusonii* | 99.79 | ATCC 35469 |
|  | **P6** |  |  |  |
| 36. |  | *Escherichia coli* | 99.13 | ATCC 11775 |
| 37. |  | *Enterococcus faecalis* | 98.8 | ATCC 19433 |
| 38. |  | *Enterococcus faecalis* | 98.35 | ATCC 19433 |
| 39. |  | *Enterococcus faecalis* | 99 | ATCC 19433 |
|  | **P7** |  |  |  |
| 40. |  | *Shigella flexneri* | 97.97 | ATCC 29903 |
| 41. |  | *Shigella flexneri* | 99.5 | ATCC 29903 |
| 42. |  | *Shigella flexneri* | 99.79 | ATCC 29903 |
|  | **P8** |  |  |  |
| 43. |  | *Escherichia fergusonii* | 99.18 | ATCC 35469 |
| 44. |  | *LFHY_s (Escherichia coli)* | 97.23 | -- |
| 45. |  | *Enterococcus durans* | 98.92 | NBRC 100479 |
| 46. |  | *Enterococcus faecalis* | 99.23 | ATCC 19433 |
|  | **P9** |  |  |  |
| 47. |  | *Escherichia fergusonii* | 99.59 | ATCC 35469 |

** Four bacterial strains are not identified.

**TABLE S3** List of bacteria isolated from adjacent biopsy samples. Classification includes Domain; Phylum; Class; Order; Family; Genus. Similarity % shows the resemblance of sequence with the nearest relative present in taxonomically united database of 16S rRNA gene sequences (Ezbiocloud.com). ATCC no. represents the top hit strain reference.

| **S.No** | **Sample** | **Classification** | **Similarity** | **Top hit** |
| --- | --- | --- | --- | --- |
|  | **A1** |  |  |  |
| 1. |  | *Escherichia* | 99.55 | B1147 |
| 2. |  | [*Shigella flexneri*](https://www.ezbiocloud.net/taxonomy?tn=Shigella%20flexneri) | 99.59 | ATCC 29903(T) |
| 3. |  | [*Escherichia fergusonii*](https://www.ezbiocloud.net/taxonomy?tn=Escherichia%20fergusonii) | 99.73 | ATCC 35469 |
| 4. |  | *Escherichia* | 99.59 | E4742 |
| 5. |  | *Escherichia marmotae* | 99.45 | HT073016 |
| 6. |  | [*Escherichia coli*](https://www.ezbiocloud.net/taxonomy?tn=Escherichia%20coli) | 99.58 | ATCC 11775(T) |
| 7. |  | [*Escherichia fergusonii*](https://www.ezbiocloud.net/taxonomy?tn=Escherichia%20fergusonii) | 99.59 | ATCC 35469 |
| 8. |  | [*Shigella flexneri*](https://www.ezbiocloud.net/taxonomy?tn=Shigella%20flexneri) | 99.45 | ATCC 29903(T) |
|  | **A2** |  |  |  |
| 9. |  | [*Shigella sonnei*](https://www.ezbiocloud.net/taxonomy?tn=Shigella%20sonnei) | 99.66 | CECT 4887(T) |
| 10. |  | [*Escherichia coli*](https://www.ezbiocloud.net/taxonomy?tn=Escherichia%20coli) | 98.65 | ATCC 11775(T) |
| 11. |  | [*Escherichia fergusonii*](https://www.ezbiocloud.net/taxonomy?tn=Escherichia%20fergusonii) | 99.73 | ATCC 35469 |
| 12. |  | [*Shigella boydii*](https://www.ezbiocloud.net/taxonomy?tn=Shigella%20boydii) | 99.32 | GTC 779(T) |
| 13. |  | [*Escherichia coli*](https://www.ezbiocloud.net/taxonomy?tn=Escherichia%20coli) | 99.37 | ATCC 11775(T) |
| 14. |  | *Streptococcus lutetiensis* | 99.79 | CIP 106849 |
| 15. |  | *Enterococcus faecium* | 99.66 | LMG 11423 |
| 16. |  | *Escherichia* | 99.73 | E4742 |
|  | **A3** |  |  |  |
| 17. |  | [*Escherichia ruysiae*](https://www.ezbiocloud.net/taxonomy?tn=Escherichia%20ruysiae) | 99.32 | OPT1704(T) |
| 18. |  | *Enterococcus durans* | 99.73 | NBRC 100479 |
| 19. |  | *Escherichia* | 99.59 | B1147 |
|  | **A4** |  |  |  |
| 20. |  | *Shigella flexneri* | 99.5 | ATCC 29903 |
|  | **A5** |  |  |  |
| 21. |  | *Escherichia fergusonii* | 99.72 | ATCC 35469 |
|  | **A6** |  |  |  |
| 22. |  | *Streptococcus lutetiansis* | 97.96 | CIP 106489 |
| 23. |  | *Enterococcus lactis* | 97.26 | BT 159 |
|  | **A7** |  |  |  |
| 24. |  | *Shigella flexneri* | 99.66 | ATCC 29903 |
| 25. |  | *Shigella flexneri* | 98.64 | ATCC 29903 |
| 26. |  | *Shigella flexneri* | 99.29 | ATCC 29903 |
|  | **A8** |  |  |  |
| 27. |  | *Enterococcus lactis* | 99.03 | BT 159 |
|  | **A9** |  |  |  |
| 28. |  | *Enterococcus lactis* | 97.87 | BT 159 |

**TABLE S4** Clinical and pathological characteristics of colorectal cancer patients

| S.No. | CRC Stage | Sex | Age | CRC Location Distribution |
| --- | --- | --- | --- | --- |
| 1 | Adenocarcinoma with signet ring cells and extracellular poor mucin | F | 46 | Ascending Colon |
| 2 | Moderately Differentiated Adenocarcinoma | F | 64 | Ileocaecal Junction |
| 3 | Moderately Differentiated Adenocarcinoma | F | 52 | Rectum |
| 4 | Chronic Inflammation in Lamina Propria | M | 53 | Rectum |
| 5 | Differentiated Adenocarcinoma | M | 36 | Rectum |
| 6 | Adenocarcinoma | M | 35 | Rectum |
| 7 | Adenocarcinoma | M | 63 | Rectum |
| 8 | Differentiated Adenocarcinoma | F | 47 | Rectum |
| 9 | Differentiated Adenocarcinoma | M | 42 | Hepatic Flexure |

**
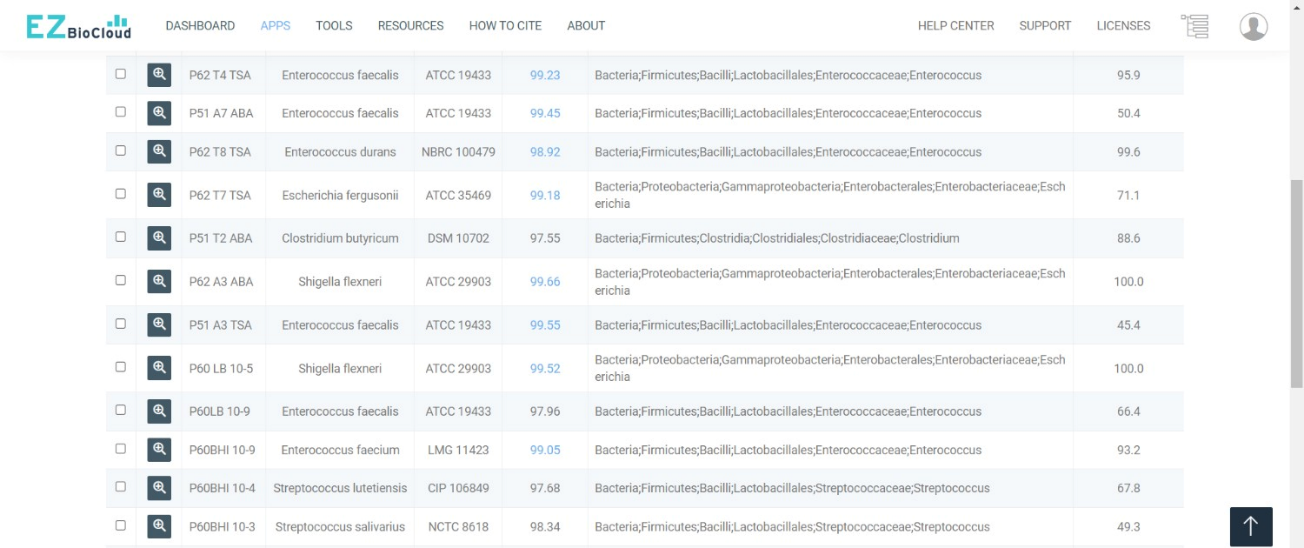
**

**SUPPLEMENTARY FIGURE 1** 16S rRNA gene-based taxonomic identification of bacterial isolates using the EZBioCloud platform.
